# Collaboration Between Health-Care Professionals, Patients, and National Competent Authorities Is Crucial for Prevention of Health Risks Linked to the Inappropriate Use of Drugs: A Position Paper of the ANSM (Agence Nationale de Sécurité du Médicament et des Produits de Santé)

**DOI:** 10.3389/fphar.2021.635841

**Published:** 2021-04-28

**Authors:** Stéphane Vignot, Pascale Daynes, Trystan Bacon, Thierry Vial, Olivier Montagne, Nicolas Albin, Joseph Emmerich, Christelle Ratignier-Carbonneil, Dominique Martin, Patrick Maison

**Affiliations:** ^1^ Collège, Agence Nationale de Sécurité du Médicament et des Produits de Santé, Saint Denis, Grand Paris; ^2^ Institut Godinot, Reims, France; ^3^ Union Francophone des Patients Partenaires, Faculté de Médecine, Bâtiment Jean Roget, La Tronche, France; ^4^ General Practitioner, Angers, France; ^5^ Hospices Civils de Lyon, Lyon, France; ^6^ General Practitioner, Collège de la médecine générale, Paris, France; ^7^ Institut Daniel Hollard, Groupe Hospitalier Mutualiste de Grenoble, Grenoble, France; ^8^ Université de Paris, Hôpital Saint Joseph Paris (Médecine Vasculaire), Paris, France; ^9^ EA 7379 EpiDermE, Paris-Est-Créteil, Créteil, France

**Keywords:** drug misuse, collegiality, anticipation, collective commitment, regulatory science

**FIGURE 1:**
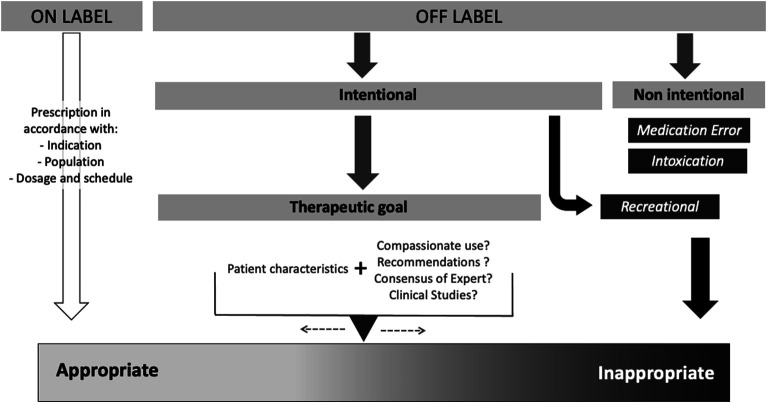
Scale of appropriate vs. inappropriate use.

All stakeholders, from patients to health-care professionals and regulatory agencies, agree to promote “proper drug use” and to fight against “misuse” (Garfinkel and Bilek, 2020; Nothelle et al., 2017). Misuse is a concept covering different situations which are summarized in Figure 1, including unintentional medication errors, poisoning, or recreational use. Such situations will not be considered in our discussion. For intentional prescriptions with therapeutic goals, defining the boundary between appropriate and inappropriate use could be challenging. Intentional off label use for therapeutic purposes can be appropriate in a specific clinical context if supported by a recommendation, consensus, or scientific data. Inappropriate use is therefore defined as unjustified intentional use, and this situation is one of the main concerns tackled by the ANSM (the French drug agency) and calls for collective action.

The appropriateness of medication use should be considered as a continuum from the conditions with the most favorable benefit–risk balance to conditions with an unfavorable balance. Yet, the more the drug use deviates from the profile defined by the summary of product recommendations, the less it is expected to benefit the individual’s health status and the more it exposes the patient to unacceptable adverse effects. From a medical point of view, a case-by-case assessment is required, taking into account all the individual patient characteristics requiring treatment and assessing comedications and comorbidities. For these reasons, medical practice requires that experimental and evidence-based data be applied to the uniqueness of the patient. This is a challenging situation explaining why the appropriate use of a drug, even with the best intention, remains a difficult choice, especially in frail populations.

Inappropriate medication use is an important source of adverse effects (Eguale et al., 2016). Very rare (<1/10,000) or rare (<1/1,000) undesirable effects, which are barely identified in clinical trials and almost impossible to detect at an individual level, are heavily represented as a health problem in the population as millions of patients are exposed to these medications over the years.

The gray area of inappropriate use is then difficult to perceive at an individual level. It is not limited to an abrupt switch between right and wrong prescriptions but could also be linked to a progressive drift in practices, leading to new risks for certain populations, in certain clinical contexts or with certain associations. It is also in this reflection that self-medication must take place. It is a direct source of inappropriate use or a factor that favors it indirectly through the risk of interactions that may not be perceptible to the prescribers.

In this context, fighting against inappropriate use and its adverse consequences is a matter of the individual relevance of the medical prescription, but global actions are also required to control them. The needs and wishes of the patient, the physician’s experience, and the population expertise of the authorities should not be opposed. Based on recent experiences, the ANSM and its expert group of advisors are advocating for a more integrated vision between the individual scale and the population approach.

In recent years, several risky health situations linked to inappropriate use of drugs have been highlighted. Consequently, health organizations such as the ANSM endorsed recommendations to limit inappropriate uses of medications. For instance, the third and fourth generations of oral contraceptives have been massively prescribed despite their higher relative risk of thromboembolic accident than the 1st and 2nd generations (Emmerich et al., 2014). This has led the ANSM to warn against the former and to recommend the latter as the first line contraceptives. Valproic acid, which has a well-known risk of teratogenicity, was commonly used by women at a childbearing age, forcing the ANSM to ban its use in certain cases (Casassus, 2017). Paracetamol is hugely used with prescriptions of high doses in France (Hider-Mlynarz et al., 2018), increasing the risk of liver toxicity. This situation led to specific measures to provide information on the dosage and to control the availability of the product in pharmacies. Inappropriate prescription of antibiotics was tackled by several awareness campaigns in France and other countries but is still a major concern for health authorities, with the emergence of extensively drug-resistant bacteria (World Health Organization, 2017).

Other initiatives have been launched by health authorities to limit the risks caused by inappropriate use of specific drugs. They are commonly based on communication strategies or regulatory action toward laboratories, but few are focused on the global issue. Some initiatives suggest focusing on particular medications and offering toolkits for prescribers (Hodgson, 2015). The most frequently selected medications according to practitioner opinions included antibiotics, proton pump inhibitors, opioids, statins, cholinesterase inhibitors, and non-steroidal anti-inflammatory drugs (Hazard et al., 2020).

These initiatives are of major importance, but may a common perception of issues related to inappropriate use still be lacking between patients, health-care professionals, and authorities? How could we build a long-term strategy beyond specific topics and crisis situations?

New perspectives should be implemented now:

- **Better anticipation**. It involves the early identification of situations where inappropriate use would lead to health risk. Such situations are characterized by the expected level of exposure in the population, the context of use and the patient profile (e.g., vulnerable populations, patients exposed to polymedication, etc.), and the pharmacological characteristics of the drug (pharmacodynamic effects, adverse effects, therapeutic margin, variability of effects, drug interactions, etc.). Taking into account these characteristics should make it possible to map risks linked to inappropriate drug use for the purpose of prevention. Detection of inappropriate use should involve all actors. Benchmarking on the use of drugs within different countries will also be a valuable tool to tackle potential misuse of drugs.
- **Better collective commitment including educational programs and co-construction of action plans with stakeholders.** Because it could be difficult to immediately assess whether the use of a particular drug is inappropriate or not, each actor needs to be responsible for its rational use. Discussion between patients and health professionals (prescribers and pharmacists) and use of prevention tools should help to identify and limit uncertain situations to decreased risks. A public debate with all the stakeholders should foster exchanges on the different perceptions and the co-construction of prevention tools in order to sensitize the entire population. The debate could lead to the development of tools to promote prevention such as computerized systems, institutional communication, or guidelines (Dalton et al., 2018; Monteiro et al., 2019). The need for new communication channels is already evident, and the best options should be chosen based on the recipients’ opinions. We can then plan specific prevention and information strategies for the known situation of inappropriate use and launch nonspecific pedagogical programs. Helping patients and professionals to understand the risks associated with the use of a drug also involves training and education (Foucaud et al., 2013). The ANSM wishes to involve academics and patient partners to co-construct training programs, in particular for patients with chronic diseases or professionals and future health professionals. The main goal of such programs should be to change patients’ and health-care professionals’ perception on the appropriate and inappropriate use of health products.

The ANSM now integrates these different perspectives in its strategy of transparency and openness to health-care professionals and the general population. In particular, patients participate as ex officio members in all expert committees, and the ANSM has integrated advisor groups including physicians and patient partners into its organization to improve co-construction of health policies on health products. Even if dialogs already existed, of course, between the agency and the stakeholders, the aim is now to organize an entanglement throughout all our reflections. This sharing of experiences and points of view, from bedside to institution, will be the cornerstone of the collective culture of appropriate use that we all wish to promote.
